# What Are the Barriers at Home and School to Healthy Eating?: Overweight/Obese Child and Parent Perspectives

**DOI:** 10.1097/jnr.0000000000000321

**Published:** 2019-09-20

**Authors:** Hee Soon KIM, Jiyoung PARK, Yumi MA, Mihae IM

**Affiliations:** 1 PhD, RN, FAAN, Professor, College of Nursing, Nursing Policy Research Institute, Yonsei University, Seoul, Republic of Korea; 2 PhD, RN, Assistant Professor, Department of Nursing, Institute of Health Science, College of Medicine, Inje University, Busan, Republic of Korea; 3 MSN, RN, Doctoral Student, School of Nursing, University of Pittsburgh, Pennsylvania, USA; 4 MSN, RN, Assistant Professor, Department of Nursing, Choonhae College of Health Sciences, Ulsan, Republic of Korea.

**Keywords:** children, feeding behavior, focus groups, pediatric obesity, South Korea

## Abstract

**Background:**

Most studies that have investigated factors influencing eating habits among obese children have focused mainly on individual or interpersonal factors and applied quantitative research methods.

**Purpose:**

This study was undertaken to identify the barriers in home and school settings that hamper healthy eating in overweight and obese children in South Korea.

**Methods:**

Focus group interviews were conducted with 15 overweight/obese children and 15 parents. A standard manual with open-ended questions was developed. Content analysis was used to identify key findings.

**Results:**

Participants were aware of the importance of home and school environments in shaping children's eating habits. Five major barriers, respectively, at home and at school emerged from the data. At home, the food preferences of parents affected the eating habits of their children. Moreover, parents worried about providing differentiated diets for siblings and about the permissiveness of grandparents toward grandsons. Furthermore, working parents preferred easy-to-prepare instant foods and said that their children ate overly quickly. At school, children cited time pressures, poor cafeteria environments, and ineffective nutrition education as barriers, whereas parents worried about inconsistent management by teachers and the unsafe food environment around the school.

**Conclusions:**

These environment-related barriers may be resolved through changes in the behavior of children, parents, and teachers as well as through the continued efforts of schools, community stakeholders, and policymakers, all of whose cooperation is essential to fostering a healthy food environment for children.

## Introduction

Childhood obesity is a serious threat worldwide (World Health Organization, 2017). In South Korea, 13.3% of children were estimated to be obese in 2016, which reflects a continuous increase over the previous decade (Korean Ministry of Health and Welfare, 2018). Obesity threatens the overall health of children by increasing the risk of new diagnoses such as asthma (Brüske, Flexeder, & Heinrich, 2014), Type 2 diabetes, high blood pressure and elevated blood cholesterol (Barton, 2012), bone and joint problems (Smith, Sumar, & Dixon, 2014), and sleep disorders (Kim, Park, Ma, & Ham, 2013). Children affected by obesity are more likely to develop various health problems during adulthood, including insulin resistance, Type 2 diabetes, cardiovascular disease (Barton, 2012), musculoskeletal disorders (Smith et al., 2014), and even cancer (Barton, 2012), and negative psychological issues, including low self-esteem and poor quality of life (Kim et al., 2013). Obese children often experience bullying and teasing in schools and are at a higher risk of social isolation (Feeg, Candelaria, Krenitsky-Korn, & Vessey, 2014). Therefore, developing more effective interventions to prevent childhood obesity has become a priority in many countries.

Obesity may be caused by an imbalance between energy intake and expenditure. This means that eating habits and physical activities, which together constitute the health behaviors that control energy balance in the body, are closely associated with becoming overweight or obese (Centers for Disease Control and Prevention, 2016). Healthy eating is a particularly critical factor influencing the weight of children. In addition, some studies have shown controlling diet to be more effective for managing childhood obesity than interventions that are designed to increase level of physical activity (Dattilo et al., 2012). Healthy eating is indicated by consuming fair amount of fruits, vegetables, whole grains, fat-free or low-fat milk and milk products, lean meats, poultry, fish, beans, eggs, and nuts, while avoiding saturated fats, trans fats, cholesterol, salt (sodium), and added sugars within daily calorie needs (Centers for Disease Control and Prevention, 2016). Previous studies have consistently reported that children who consume high-fat or high-sugar foods and fast foods face a higher risk of being overweight or obese (Dattilo et al., 2012) and that undesirable eating habits such as binge eating, hurried eating, and eating an unbalanced diet are also closely related to childhood obesity (Savage, Fisher, & Birch, 2007). This means that children who are overweight or obese may share unique eating environments with their parents that differ from children who are at a healthy weight. Thus, understanding what children and parents perceive as barriers to healthy eating in children is highly important to developing healthy eating habits in children and, ultimately, to preventing childhood obesity.

The importance of adopting an ecological approach to the development of health promotion programs for children has recently been emphasized in the literature (Korin, 2016, pp. 16–17). This approach is premised on giving full weight to the fact that human behavior is affected by various environmental factors as well as individual factors such as personal attitude and knowledge (McLeroy, Bibeau, Steckler, & Glanz, 1988). As this implies, eating habits, as a health behavior, are affected not only by individual characteristics but also by related environmental characteristics in the home, school, and community. A qualitative study by Park, Kang, Lawrence, and Gittelsohn (2014) involving parents and teachers showed, in the South Korean context, that home environment was the most significant factor influencing youth eating habits and that poor nutrition education in school was associated with lower vegetable intake among children. In addition, a qualitative study by Banna, Buchthal, Delormier, Creed-Kanashiro, and Penny (2016) involving adolescents in Peru found that various ecological factors at the individual, social and physical environmental, and macrosystemic levels affected eating habits. Thus, using an ecological approach to explore the environmental factors that constitute barriers to the eating habits of obese children is important.

Parents and family members, classified as “interpersonal factors” in the ecological approach, are the most important environmental factors affecting the formation and maintenance of the eating habits of children (Savage et al., 2007). In particular, it is known that the attitudes and knowledge of parents with regard to good eating habits greatly influence the eating habits of their children, which supports the position of parents as critical role models for their children (Savage et al., 2007). Parental food preferences also determine the type and quality of food that a child consumes in the home (Scaglioni, Arriza, Vecchi, & Tedeschi, 2011).

In addition to parents and family, schools, as an “organizational factor” in the ecological approach, have a significant impact on the eating habits of children. Because the school environment where children live, learn, and play has such great potential to impact their eating habits, identifying risks to those habits that emerge at school and implementing long-term, well-rounded interventions to address these risks are important (Yang et al., 2015). In addition, a large portion of the total daily food intake of children occurs at school, making school one of the most suitable places to teach and train children about healthy eating (Vio, Fretes, Montenegro, González, & Salinas, 2015). Moreover, school principals and teachers are well placed to reach both children and parents to aid in the implementation of nutritional guidelines and the regulation of food and beverages, thus fostering an environment that encourages healthy eating habits (Leines, Gold, & Van Offelen, 2014). In one study in the United States, children reported increased consumption of fruits and vegetables when school teachers reinforced nutrition education in their classes (Leines et al., 2014).

However, despite the importance of environmental factors in the eating habits of children, most of the studies on the factors that influence eating habits in obese children in South Korea have focused on individual or interpersonal factors and used quantitative research methods (Yoo, Yoon, & Ha, 2016). Only a limited number of studies have used qualitative methods to investigate the environmental factors that may affect these eating habits (Park et al., 2014) and those that have neither provided sufficient information to identify the needs of children or their parents nor gathered participant opinions regarding the development of effective interventions. Therefore, the goal of this study was to conduct focus group interviews with overweight and obese Korean children and their parents to gather information about self-perceived barriers to healthy eating habits in home and school settings. The findings may be used to inform the development of multilevel interventions for preventing childhood obesity.

Overweight and obese children and their parents were recruited as participants. The main assumption of this study was that these children and their parents face certain shared barriers to healthy eating. In terms of focus groups, the parents were divided into housewives and working parents, as parental employment status is an important factor affecting the eating habits of children (Bauer, Hearst, Escoto, Berge, & Neumark-Sztainer, 2012). This study posed the following two research questions: (a) What are the interpersonal and organizational barriers to healthy eating that are perceived by overweight and obese children and their parents? (b) Are there significant differences in these perceived barriers between housewives and working parents?

## Methods

### Design and Participants

This qualitative research study conducted focus group interviews with overweight and obese children and their parents to identify barriers to healthy eating in home and school settings. Unlike quantitative research methods, qualitative research methods may be used to elicit the perceived barriers of children and parents to adopting healthy eating behaviors. This study aimed to identify the barriers to adopting healthy eating habits of overweight or obese children and their parents. Focus group interviews were used to obtain opinions regarding personal or group feelings, perceptions, and opinions while observing the underlying group dynamic (Krueger & Casey, 2014). The shared experiences of the children of being overweight or obese as well as the shared experiences of the parents of caring for overweight or obese children were examined. The focus group interviews were expected to elicit the uniquely shared environments, focusing on the perceived barriers of the participants to healthy eating.

The ecological model was used as the conceptual framework to guide the development of interview questions and the data analysis (McLeroy et al., 1988). Because this study was more interested in exploring environmental barriers to healthy eating, it focused on environmental influences, such as interpersonal and institutional factors, instead of personal characteristics. In this study, “interpersonal barriers” are defined as barriers related to family, and “institutional barriers” are defined as barriers related to school. Study participants included overweight and obese children between 7 and 11 years old who were participating in a Y Health Coaching Program and their parents. The program was supported by the Korean Ministry of Health and Welfare, which regularly provides physical activity and nutrition education for obese children. Two focus groups with 15 overweight and obese children and two focus groups comprising their parents were conducted. The children and their parents were respectively divided into two groups based on parental employment status (Table 1).

**TABLE 1.**
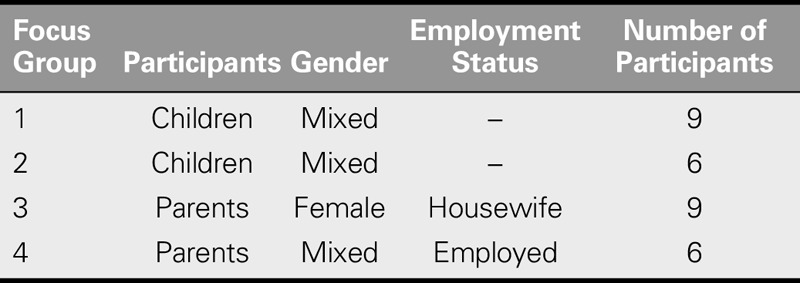
Overview of the Focus Groups (*N* = 30)

### Development of the Study Manual

A study manual for the focus groups was developed in accordance with the protocol established by the Identification and Prevention of Dietary- and Lifestyle-Induced Health Effects in Children and Infants project team, which is a multicenter European collaborative project to prevent childhood overweight and obesity (Haerens et al., 2009). The protocol includes guidelines for focus group study, covering recruitment, setting, moderator's role, and open-ended questions on eating behaviors and physical activity for children and their parents. For instance, regarding the guidelines for the moderator, it introduces an approach to preparing, leading, and closing the focus group sessions and provides an example that is applicable to actual focus group situations. The authors of this study were authorized by the Identification and Prevention of Dietary- and Lifestyle-Induced Health Effects in Children and Infants project team to use the official protocols, which were subsequently translated. The interview questions were modified to adhere to Korean cultural norms. For example, questions related to satisfaction with institutional food service at school, including the role of teachers, service environments, and diets, were added. The interview questions for children addressed food preferences, food availability, and food-related rules at home and at school as well as types of education at school. The interview questions for parents addressed barriers to their children's healthy eating at home and school, availability or rules at home and at school, and the role of the school. If the answers of participants related to barriers to healthy eating, the researchers asked additional, probing questions. Pilot interviews were conducted with two school-aged children and their parents to confirm their understanding of the interview questions, with the findings resulting in the modification of several interview questions to reflect their opinions or suggestions.

### Data Collection Procedures

This study was approved by the institutional review board of the researcher's university (IRB no. 2012-0012). Focus group interviews were conducted from June to September 2012. Two weeks before the interview, recruitment letters were sent to the parents of potential participants, all of whom were enrolled in a Y Health Coaching Program. Each group consisted of six to nine children or parents, with group assignments based, as previously noted, on parental employment status. Parents completed a brief personal demographics survey before the interview. The children and parents were interviewed for 60 and 90 minutes each, respectively. Informed consent was obtained from all of the participants, who were informed that participation was voluntary and that all interviews would be recorded. A moderator asked the questions based on the standardized study manual and encouraged participants to speak freely and comfortably. A co-moderator recorded key information and viewpoints in the form of field notes during the discussion. The participants received a $30 gift card in compensation for their participation.

### Data Analysis

Descriptive statistics were calculated to obtain the general characteristics of the participants, using Predictive Analytics Software Statistics 18.0. All of the recorded interviews were transcribed verbatim, and thematic analysis was used to identify key findings. Two researchers reviewed and analyzed the transcripts independently. Next, the data were abstracted into a matrix developed based on the ecological model to identify themes. Results were compared between the two researchers, with any differences reviewed by a third researcher to reach consensus. Throughout this process, researchers tried to understand the ideas of the participants and the meaning in their responses by listening to the recorded files and reviewing the transcriptions repeatedly.

The validity of the qualitative data was confirmed based on credibility, transferability, dependability, and confirmability (Lincoln & Guba, 1985). To ensure credibility, researchers allowed participants to speak freely and refrained from asking leading questions. Moreover, researchers tried to exclude subjectivity and prejudice from their data analysis. The verbatim oral statements of participants were retained, and meaningful concepts, phrases, subcategories, and categories were extracted to ensure transferability. The results were modified several times by researchers over time to make sure that the results were well reflected, which ensured dependability and confirmability.

## Results

### General Characteristics of Participants

The mean age of the children who participated in this study was 9.6 (*SD* = 1.30) years. Two thirds were boys (66.7%), and one third were girls (33.3%). On the basis of the age- and gender-specific reference growth chart for Korean children (Korea Centers for Disease Control and Prevention, The Korean Pediatric Society, 2007), 13.3% of the child participants were overweight and 86.7% were obese. The mean age of parents was 39.9 (*SD* = 5.5) years, and 93.3% were female. On the basis of the body mass index classification standard for Asians (Korea Centers for Disease Control and Prevention, The Korean Pediatric Society, 2007), 26.7% of the mother participants were overweight and 40.0% were obese. Slightly less than half (46.7%) of the parents were high school graduates, and 40% were employed (Table 2).

**TABLE 2.**
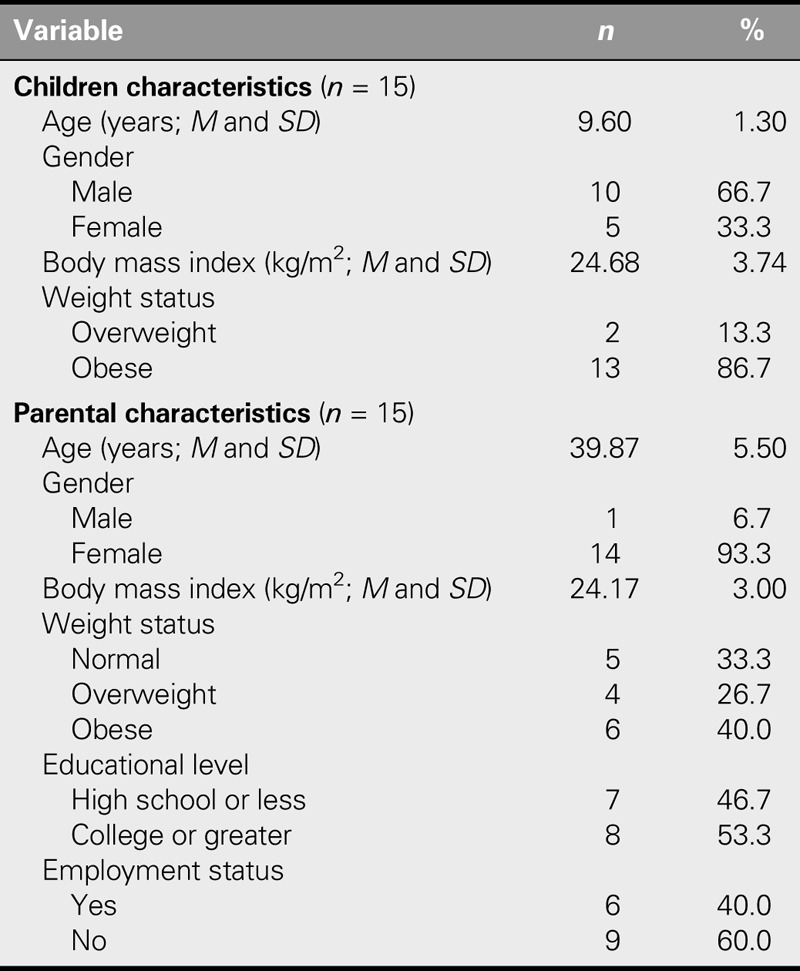
General Characteristics of Participants (*N* = 30)

### Barriers Influencing the Eating Habits of Overweight and Obese Children

**Figure 1.**
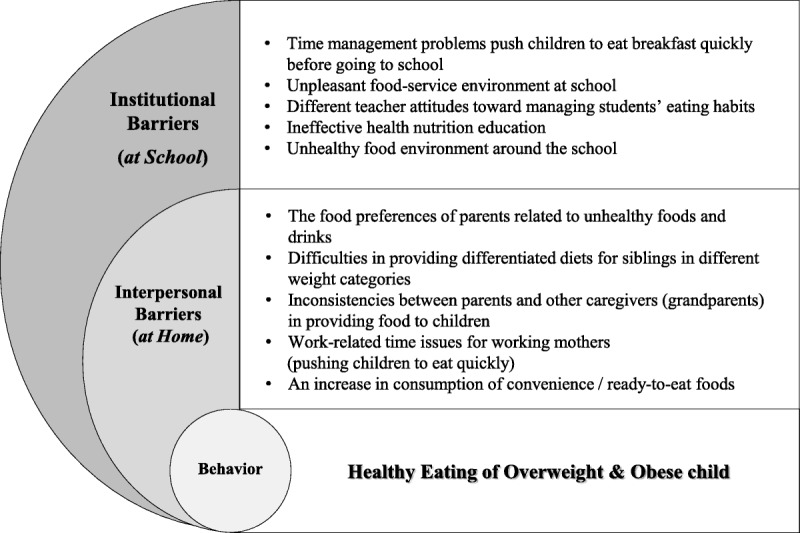
Barriers at home and school to managing healthy eating.

The findings were segregated into interpersonal and institutional levels based on the ecological model. Five major interpersonal barriers and five major institutional barriers were identified (Figure 1). Significant differences in interpersonal barriers were found between housewives and working parents.

### Interpersonal Barriers

#### The food preferences of parents related to unhealthy foods and drinks

The parents of overweight or obese children expressed that their food preferences negatively affected their management of their children's eating habits at home.

*My husband and I drink a lot of soft drinks at home. Naturally, it has become a habit for my child to drink soft drinks instead of water.* (Housewife)

*I feel so guilty when it comes to ramen, since I like ramen very much. I eat it once or twice a week. I think I should cut back on it.* (Working parent)

#### Difficulties in providing differentiated diets for siblings in different weight categories

The parents with multiple children in various weight categories expressed experiencing difficulties in providing differentiated diets at home for their children.

*Since I have three children, it's rather difficult for me to give snack foods to my second child and my youngest but to tell my oldest to eat only fruits.* (Housewife)

*When it comes to foods like ham, I give more to my older daughter and less to my younger daughter. Then, my younger child would complain, ”Mom, why do you only hate me...?” I could manage my children's diet at the same time if they were both overweight. But, it's hard for me to manage their diet, because one of them is fat and the other is skinny.* (Working parent)

#### Inconsistencies between parents and other caregivers (grandparents) in providing food to children

The parents highlighted inconsistencies between parents and other caregivers in enforcing restrictions on unhealthy food for their overweight or obese children.

*Even though I say the children are not allowed to have cookies, my mother-in-law insists that she wants to get the kids snack foods and, in fact, tells the children to choose whatever they want when we go the supermarket together with them.* (Housewife)

*Ramen is also a problem at my house. My mother-in-law makes ramen for the kids behind my back so often.* (Working parent)

#### Work-related time issues for working mothers (pushing children to eat quickly)

The working parents, but not housewives, of children who are overweight or obese said that they have the tendency to push their children to eat quickly.

*I think I've gotten into a habit of saying ”quickly, quickly” because my husband and I both work. Whenever my child eats quickly, she has to eat again before she feels full. As a result, I feel that she naturally ends up eating faster and more.* (Working parent)

#### An increase in consumption of convenience or ready-to-eat foods

In addition, the working parents (but not housewives) of children who are overweight or obese said that they have increasingly provided convenience foods without considering nutrition values or ingredients.

*You know, foods like mini-meatballs,…those that are rather easy to make. I end up making them frequently because I am tired as well, although I know that's a problem.* (Working parent)

*I end up feeding my kids one-dish foods at home because we are a dual-career family.* (Working parent)

### Institutional Barriers

#### Time management problems push children to eat breakfast quickly before going to school

Children who are overweight or obese stated that they have to eat their breakfast quickly because of the limited time available to get ready for school.

*I have to go to school in the morning, so I just mix rice in soup and eat quickly.* (Housewife's child)

*I eat slowly during dinner time, but I don't have much time in the morning, and end up eating fast. I'm in such a hurry to go to school.* (Child of working parents)

#### Unpleasant food service environment at school

Most of the children and parents were satisfied with the food service provided at schools.

*(“How are the school meals?”) I like everything about them.* (Housewife's child)

*They taste good.* (Child of working parents)

*The school meals are much better than what we have at home. They are always healthy foods like vegetables. In terms of nutrition, the school meals are far better than what we eat at home.* (Working parent)

However, they pointed out flaws in the physical food service environment at school such as lack of space, which prevents the children from enjoying the food in a relaxed atmosphere.

*(“How is the school cafeteria?”) There are too many people. It's so noisy.* (Child of working parents)

*[They] eat quickly. Since the school also has a designated cafeteria, the students have to give room for the next class or grade. So, they have to eat quickly at school as well.* (Working parent)

#### Different teacher attitudes toward managing students' eating habits

The parents expressed concern over the significant effect of homeroom teachers' attitude on their children's eating habits.

*There is a huge difference between teachers. There are some teachers who are very good at teaching the kids about eating habits…. But then, things change when they are assigned to another teacher in the next grade…. As one student’s mom said, a child’s eating habits can change six times depending on who the teacher is….* (Housewife)

*If the teacher has an awareness of good eating habits, he/she thoroughly manages the kids. Otherwise, the children can be neglected again and left to eat whatever they want or even throw away their cafeteria food.* (Housewife)

#### Ineffective health nutrition education

Most of the children and parents stated that the nutrition education provided at school is ineffective. As part of their improvement recommendations, they emphasized the importance of providing continuous nutrition education that takes into account children's interests and applications to real life.

*(“Have you learned about things like healthy food?”) In P.E. class.* (Housewife's child)

*I've learned about it in health class….* (Housewife's child)

*(“Were you able to get a lot of information from the class?”) I already knew most of the information.* (Housewife's child)

*They don't teach us in detail.* (Housewife's child)

*I just want it to be over.* (Housewife's child)

*When it comes to education, I think the school should constantly teach the children that “a slice of pizza is a bowl of rice.” My child didn't eat pizza for a while. The school shouldn't teach the kids about this food having this much calories and that food having that much calories. Instead, they should teach that this food is the same as a bowl of rice and that, if you want to lose weight, you should ride a bike for thirty or forty minutes. I am saying that they should teach the kids in a straightforward manner.* (Housewife)

#### Unhealthy food environment around the school

The parents stated that unhealthy food is easily bought and consumed around the school.

*(“Are there places around the school where you can eat?”) There are so many places around the school. There are even snack bars*…. (Housewife's child) *(“What do they have there?”) Unsanitary food like…Apachi and Apollo, Zzonzzoni, Minimong, Nemo snack…uhmm…and beer-shaped candy that makes your lips turn blue….* (Housewife's child)

*Sometimes, the kids who have money buy other kids unsanitary food at the stationary stores. These places have so many cheap snacks…. Just [an amount of money equivalent to 10 cents US]….* (Housewife)

*At the stationary stores. The kids eat unhealthy food like taffies that are high in calories.* (Housewife)

## Discussion

### Interpersonal Barriers

The results of this study reveal that the food preferences of parents for items such as carbonated beverages and ramen had a negative impact on the eating habits of their overweight and obese children. As the children's role models, parents are a major environmental factor affecting the formation of their children's eating habits (Savage et al., 2007). When parents prefer high-fat, high-calorie food, children lose the chance to eat a variety of dishes and to control the food that they eat themselves (Scaglioni et al., 2011). It is necessary to educate parents continuously about this aspect of their role and about the importance of managing their own eating habits to manage the eating habits of their overweight or obese children.

Parents also expressed difficulties with providing differentiated menus or foods to their children in multiple weight categories. They tended to allow their children who were not obese to eat high-fat diets or snacks, which gave the impression to their obese children of being discriminated against. Forcing a child to eat excessively or restricting his or her diet hinders the formation of healthy eating habits (Scaglioni et al., 2011). Moreover, the feeling of being discriminated against at home may increase the obesity-related stress perceived by the child, which may lead to negative eating habits such as binge eating and overeating. Thus, it is important that parents provide a healthy diet consistently to all of their children rather than allowing different diets based on weight category. In addition, with regard to the permissive attitudes of grandparents toward their grandchildren's eating habits, which was another worry of parents, previous studies consistently report that permissive parenting attitudes affect children's eating habits and obesity negatively (Hennessy, Hughes, Goldberg, Hyatt, & Economos, 2012; Savage et al., 2007). In Korea, as grandparents often play the role of primary caregivers, permissive and inconsistent (grand)parenting may undermine the efforts of parents and make it difficult for children to form good eating habits. Thus, education is also needed for grandparents to help them achieve consistent, encouraging, and supportive parenting attitudes related to the eating habits of their grandchildren.

#### Differences among barriers perceived by housewives and working parents

In this study, the focus group interviews were conducted separately for housewives and for working parents, as parental employment status has been shown to affect the eating habits of children (Bauer et al., 2012). This study found that working parents tended to encourage their children to eat quickly and preferred to prepare instant food because of the limited time available for meal preparation. Furthermore, parents frequently multitasked during mealtime. In previous studies, working mothers were found to finish meals quickly so that they could complete other tasks, a habit that was found to be associated with unhealthful diets and that may contribute to obesity and related health problems (Fulkerson et al., 2011). In addition, children of working parents eat out more frequently and consume more food and calories as a result (Bauer et al., 2012). Therefore, we suggest providing daily nutrition guidance using social network services such as KakaoTalk, a popular SNS in South Korea, to facilitate family engagement in planning and cooking quick and healthful meals.

Furthermore, obesity management programs should be developed differently based on the employment status of parents.

### Institutional Barriers

In this study, the children participants stated that they were expected to eat breakfast in a hurry because school started early in the morning. Schools in South Korea rarely provide breakfast, so children often eat in a hurry, go to school late, or miss breakfast altogether. Yang et al. (2015) showed that the main reason for missing breakfast in South Korea was lack of time because of the early starting time of school. Breakfast plays an important role in maintaining good health in children and affects their school life. The U.S. government instituted a breakfast program for students from low-income families, with participating students showing lower rates of absence, better concentration in the classroom, and better school adjustment (Wilder Research, 2014). School and community health policymakers must acknowledge the negative effects of skipping breakfast and make structural efforts to improve the ratio of breakfast consumption.

Most children and parents in this study were satisfied with the nutritional value and quality of the meals being served at school. However, they were dissatisfied with the dining environment. Students ate in a hurry because of cramped dining facilities and the short time allowed for lunch. Respondents to the Korean 2014 National School Meal Satisfaction Survey complained about eating spaces being excessively noisy and waiting times being overly long (Yang et al., 2015), results that are similar to those of this study. The school dining environment affects the formation of eating habits as significantly as the type of food served. An unhealthy dining environment tends to encourage students to think of school lunch as an expected routine or even a chore that must be done as part of school life rather than a pleasant experience (Wilder Research, 2014). Therefore, schools should implement plans such as increasing the number of dining facilities and setting appropriate rules during mealtime.

Participants in this study reported that different teachers exhibited different diet management attitudes and instructed students differently. They also emphasized the important role of teachers in the eating behavior of their children at school. Teachers are important role models who greatly affect the formation of children's eating habits. Previous studies have shown that students are more likely to be exposed to unhealthy foods as a result of the actions of teachers who drink soft drinks at school or give candy or pizza to students as rewards or incentives (Arcan et al., 2013). In addition, the level of knowledge and guidance that teachers provide on eating habits was found to correlate highly with healthy eating habits in students (Vio et al., 2015). As important role models, teachers should work to enhance the nutrition-related knowledge of their students and help them establish healthy eating habits—ideally through formal nutrition education programs.

In addition, the children and parents in this study mentioned that the content of nutrition education at school was superficial, repetitive, and difficult to apply in real life, making children feel bored. Nutrition education in elementary school plays an important role in forming good eating habits and affects future health and health behaviors (Vio et al., 2015). In South Korea, dieticians are responsible for managing school meals as well as teaching nutrition education, making it difficult for them to find time to plan lessons and teach effectively (Oh et al., 2016). Previous studies that evaluated the satisfaction of elementary school students with their nutrition education in South Korea also indicated that nutrition education was boring and difficult to apply in practice because it was lecture-based education instead of an experiential activity (Yoo et al., 2016). Therefore, nutrition education in school should be expanded and developed using practical education programs that consider students' interests and motivations such as cooking and gardening classes.

Most South Korean elementary schools are located in densely populated residential areas. Thus, students typically go to school on foot. Consequently, low-quality foods that easily attract the attention of students are often sold around schools, and children are exposed to low-quality food that is high in sugar, fat, and sodium. To ameliorate this problem, the South Korean government has designated the 200-m radius around all schools as “Green Food Zones,” where sales of high-calorie, junk, and high-caffeine foods are regulated (Kim, Jung, & Kim, 2016). However, government field surveys have found that stationery stores in these zones continue to sell unhealthy food and junk food that often do not indicate country of origin and may contain tar or be contaminated with *E. coli* (Kim et al., 2016). Therefore, school education should be strengthened to give children knowledge about and promote good attitudes toward healthy foods. Furthermore, the cooperation and efforts of the local community are essential for creating a healthy food environment around the school. This effort should include requiring higher penalties for selling inappropriate foods in Green Food Zones.

### Limitations

The results of this study were drawn from a limited number of participants living in a metropolitan area. Thus, the generalizability of results to other populations within and outside Korea is limited. In addition, this study investigated only the perspectives of children and their parents. The views of other stakeholders, including teachers and local and national policymakers, may not align with those of the participants. Further studies including these groups are needed. Finally, future studies should compare the differences in perspectives between children and their parents and investigate the barriers at school that relate to child–teacher relationships.
